# Droplet Merging on a Lab-on-a-Chip Platform by Uniform Magnetic Fields

**DOI:** 10.1038/srep37671

**Published:** 2016-11-28

**Authors:** V. B. Varma, A. Ray, Z. M. Wang, Z. P. Wang, R. V. Ramanujan

**Affiliations:** 1 School of Materials Science and Engineering, Nanyang Technological University, 639798, Singapore; 2 Singapore Institute of Manufacturing Technology, 71 Nanyang Dr, 638075, Singapore

## Abstract

Droplet microfluidics offers a range of Lab-on-a-chip (LoC) applications. However, wireless and programmable manipulation of such droplets is a challenge. We address this challenge by experimental and modelling studies of uniform magnetic field induced merging of ferrofluid based droplets. Control of droplet velocity and merging was achieved through uniform magnetic field and flow rate ratio. Conditions for droplet merging with respect to droplet velocity were studied. Merging and mixing of colour dye + magnetite composite droplets was demonstrated. Our experimental and numerical results are in good agreement. These studies are useful for wireless and programmable droplet merging as well as mixing relevant to biosensing, bioassay, microfluidic-based synthesis, reaction kinetics, and magnetochemistry.

Droplet microfluidics (DMF)^1^,^2^ is a versatile tool for the manipulation of matter^3^,^4^,^5^ on a Lab-on-a-Chip (LoC) platform. LoC applications of DMF require droplet manipulation in order to perform operations such as droplet merging, mixing, breakup, and sorting. Droplet manipulation on a LoC platform has been used for the synthesis of novel materials^6^ relevant to biosensing^7^, three-dimensional (3D) cell culture^8^, photonic crystals^9^, Janus structures^10^, anisotropic particles^11^, functional polymeric structures^12^,^13^,^14^, controlled encapsulation for drug delivery^15^ and multidimensional optical barcoding^16^. Specifically, droplet merging and mixing on LoC platform has been used for a broad range of biomedical applications^17^ such as bioassays^18^,^19^, biomaterials^20^, single cell analysis^21^, cell sorting^22^,^23^, population transcriptomics^24^, disease detection^25^, and diagnostics^26^. Merging and mixing of droplets on a LoC platform can also lead to miniaturized volume conditions (nl to pl), reduced operation time and several fold increase in screening of chemical reactions^27^, which opens a new domain of DMF chemistry^28^,^29^, drug discovery applications^30^,^31^,^32^, high-throughput molecular genetics^33^, interfacial studies^34^ and ‘on water reaction’^35^.

In previous reports, various strategies were utilized to merge droplets e.g., hydrodynamic flow focusing^36^, surface acoustic waves^37^, dielectrophoresis^38^, surfactant-hydrodynamic flow focusing^39^, droplet velocity-lipid concentration^18^, electrorheological fluids^40^, cavitation bubble^41^, electro-coalescence^42^ and liquid phase flow^43^. Most of the above strategies require one or more of the following: direct contact with the liquid, complex microfabrication techniques, changes in the channel geometry, or changes in the flow rates.

Hence, contact-free, wireless and programmable manipulation capabilities for droplet breakup, merging and mixing will be advantageous. In literature, generally a combination of non-uniform magnetic field and ferrofluid droplets (FFDs) was used to demonstrate various aspects of LoC operations, such as: (i) FFD breakup by the non-uniform magnetic field of a permanent magnet in a microfluidic Y junction^44^, flow focusing devices^45^,^46^, a straight channel^47^ and at a T-junction^48^. (ii) Nguyen *et al*. investigated FFD size control and formation by the field of a permanent magnet; one^49^ and two^50^ dimensional manipulation by planar coils and kinematics, deformation by two pairs of planar coils^51^, and FFD actuation by a coil-magnet combination^52^. (iii) Chen *et al*. investigated the effect of the rotational field on self-assembly of FFD^53^ and ordered FFD formation^54^. (iv) Di Carlo *et al*., demonstrated magnetic droplet generation rate, and size control by a magnetically driven technique, consisting of a gradient magnetic field of a permanent magnet^55^. (v) Sanders *et al*. demonstrated magnetic transport-release^56^ by a permanent magnet.

Recent reports of the use of the magnetic field to perform FFD merging include: (i) Xiao *et al*.^57^ used spin torque oscillator to demonstrate stationary droplet merging by a combination of applied current and magnetic field. However, those studies were not performed on LoC platform; (ii) Ahmadi *et al*.^58^ reported a magnetohydrodynamic method for actuation and merging of millimetric droplets. The role of the magnetic force on FFD manipulation was also investigated; (iii) Teste *et al*.^59^ demonstrated a ferromagnetic rail based manipulation system to control ferrofluid droplets motion and merging.

From the literature it is evident that the integration of non-uniform magnetic fields on LoC platform is limited by (i) requirement of a large magnetic field gradient; (ii) high sensitivity of the FFD control to the position of the gradient along the microchannel^60^; (iii) larger size of permanent magnets than the microchannel size; (v) lack of programmable operations with permanent magnets; (vi) complex designs, fabrication techniques required for micro-coil integration with the microfluidic chip; and (vii) magnetic force is limited on LoC platform due to smaller FFD size, hence manipulation at lower magnetic fields is difficult and challenging. These factors limit the advantages of using magnetic fields for LoC droplet manipulation. The above challenges can be addressed by a combination of *magnetic fluids* and *uniform magnetic fields,* which offers a *wireless, programmable* and *remote* method to perform LoC operations. In the literature uniform magnetic field was used on LoC platforms to investigate ferrohydrodynamic instabilities in uniform magnetic field^61^,^62^, FFD formation^63^,^64^, non-linear deformation of FFD^65^, magnetic trapping of bacteria^66^, magnetofluidic mixing^67^, and spreading^68^.

Uniform magnetic fields can be used for contact-free, wireless, programmable and precise manipulation of magnetic fluid droplets. However, the use of the uniform magnetic field for the wireless and programmable merging of moving magnetic fluid droplets on a DMF based LoC platform has not yet investigated in detail.

Hence, the present work reports for the first time, *uniform magnetic field induced* merging of moving droplets on a LoC platform. The merging of ferrofluid droplets at various magnetic field strengths and flow rate ratios were investigated. The control of ferrofluid droplet merging was demonstrated experimentally and numerically. A micromagnetofluidic numerical model was developed to investigate the process of droplet merging. The merging of colour dye + ferrofluid composite droplets was demonstrated. These studies can be useful for wirelessly controlled merging, mixing of droplets, Janus particle formation, reaction kinetics, and biosensing.

## Results and Discussion

**Figure 1:**
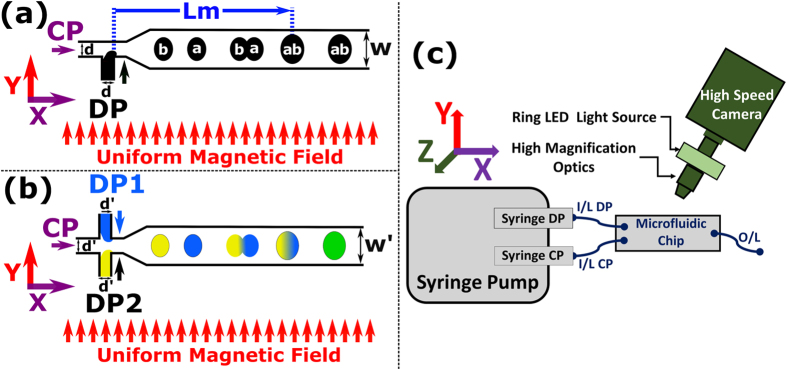
Schematic of uniform magnetic field induced merging of (**a**) Ferrofluid droplets (150 μm channel height). Under the influence of uniform magnetic field H, droplet ‘a’ (with velocity Va) merges with droplet ‘b’ (with velocity Vb), yielding a droplet ‘ab’ (with velocity Vab). (**b**) Colour dye + magnetite (ferrofluid) composite droplets (100 μm channel height). (**c**) Schematic of micromagnetofluidic setup^68^,^69^ (i) microfluidic droplet generation along the CP flow in the x-direction, (ii) magnetic field, H along y-direction (iii) high-speed imaging. The high-speed camera is mounted in the z-direction, perpendicular to both the CP flow and magnetic field direction. Inlet and outlet of the microfluidic chip are denoted by I/L and O/L, respectively. CP = continuous phase, DP = Dispersed Phase, d = 150 μm, W = 500 μm, d’ = 100 μm and W’ = 300 μm (not to scale). Please see Table 1 for ferrofluid properties.

Our experimental studies of uniform magnetic field induced droplet merging can be divided into (a) Generation and merging of ferrofluid droplets under applied uniform magnetic field (H) and (b) Generation and merging of colour dye + ferrofluid composite droplets. Two designs were used for droplet generation (Fig. 1), using two immiscible phases: oil as the continuous phase (CP) and water-based ferrofluid (please see Table 1 for ferrofluid properties) or water based dye solution as the dispersed phase (DP). Uniform magnetic fields were applied perpendicular to droplet flow, and high-speed imaging was performed using our micro-magnetofluidic setup^68^,^69^.

Our studies start with the generation of droplets at various flow rate ratios, Qr. The effect of flow rate ratio and an applied uniform magnetic field on the droplet size (before merging) is described in the first subsection. The second subsection describes magnetically induced merging of ferrofluid droplets at various flow rate ratios. The process of merging is then elaborated by investigating the variation of droplet velocity with time. The experimental setup, notations, and definitions are described in the *methodology section*.

### Generation of Ferrofluid Droplets at Different Flow Rate Ratio

**Figure 2:**
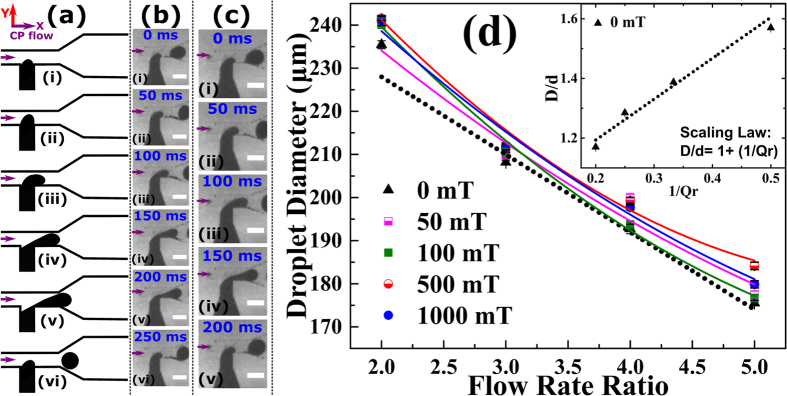
Generation of ferrofluid droplets in a uniform magnetic field. (**a–c**) Droplet generation at the flow rate ratio 2 (Q2). (**a**) Schematic. (**b,c**) Experimental micrographs at (**b**) H = 0 mT and (**c**) H = 500 mT, scale bar = 250 μm. (**d**) Droplet size vs flow rate ratio (Qr) at magnetic field H = 0, 50, 100, 500, 1000 mT. Solid lines denote polynomial fits and the dotted line shows a linear fit. The inset shows the graph for the scaling law D/d = 1 + 1/Qr^70^ at H = 0mT, for droplet diameter D, channel width d and flow rate ratio Qr = Q_cp_/Q_dp_. The purple arrow indicates the direction of the CP flow (x-direction). The magnetic field is in the y-direction. Please see Table 1 for ferrofluid properties.

Ferrofluid droplets were generated using a T-junction configuration on a LoC platform (Fig. 1). Droplet formation for all of our experiments is in the ‘*squeezing’* regime since the capillary number^70^ (Ca = η_cp_v_cp_/σ) is less than 10^–2^, where η_cp_, v_cp_ and σ are the viscosity of the CP, velocity of the CP and surface tension, respectively. At a constant DP flow rate, droplet formation proceeds in the following steps (Fig. 2a): (i) The ferrofluid enters the main channel and forms a paraboloid. (ii) A neck is developed as the paraboloid grows in size. (iii) The neck elongation starts as the paraboloid advances. (iv) The paraboloid confines the flow of the CP, resulting in the ‘squeezing of the neck’ due to increased upstream pressure. (v) The squeezing of the neck proceeds at a rate which is proportional to the flow rate of the CP, this decides the droplet size. (vi) Finally, the neck breaks and the droplet detaches from the DP stream. When a magnetic field was applied (H = 500mT, Fig. 2c) the droplet generation time was 200 ms, which was smaller than the generation time of 250 ms without a magnet field (H = 0mT, Fig. 2b). This decrease in droplet generation time is due to the additional force contributed by the applied magnetic field (i.e., the magnetic volume force).

At zero magnetic field, a linear decrease in droplet size was observed with increasing flow rate ratio Qr (Fig. 2d). Since the droplets are formed in the squeezing regime (Ca < 10^−2^) by the T-junction, the scaling law D/d = 1 + 1/Qr is followed at zero fields for droplets with size D, channel width d and flow rate ratio Qr = Q_cp_/Q_dp_^70^. With increasing magnetic field, an increase of ~15 μm in droplet size was observed. This increase was caused by the magnetic volume force, which increases the DP flow, leading to increased droplet size. However, this general behaviour was not followed for 1000 mT, where a decrease in the droplet size was observed compared to the size at 500 mT. The non-uniform component of magnetic field ≤1%, implying (i) for a field of 500 mT nonuniformity of ±5mT and (ii) for 1000 mT, nonuniformity of ±10 mT. With the increasing uniform magnetic field increases the elongation of the ferrofluid droplets and hence increases the surface tension of the ferrofluid droplets^71^,^72^,^73^,^74^. The observed decrease in the droplet sizes at 1000mT than that for 500 mT is the result of increased surface tension and also non-uniformity of ±10 mT.

### Droplet merging

**Figure 3:**
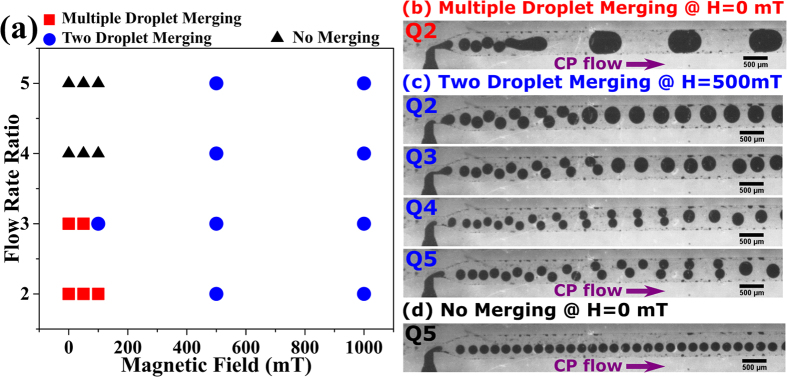
(**a**) Droplet merging map depicting various droplet merging regimes. (**b–d**) Experimental micrographs (scale bar = 500 μm) for droplet merging regimes: (**b**) Multiple droplet merging (**c**) Two droplet merging (**d**) No merging. Q2, Q3, Q4 and Q5 denotes flow rate ratios. Please see Table 1 for ferrofluid properties and Table 2 for notation. The purple arrow indicates the direction of the CP flow (x-direction). The magnetic field is in the y-direction. A video S2 is available in the supplementary files, showing the experimental droplet merging regimes.

**Figure 4:**
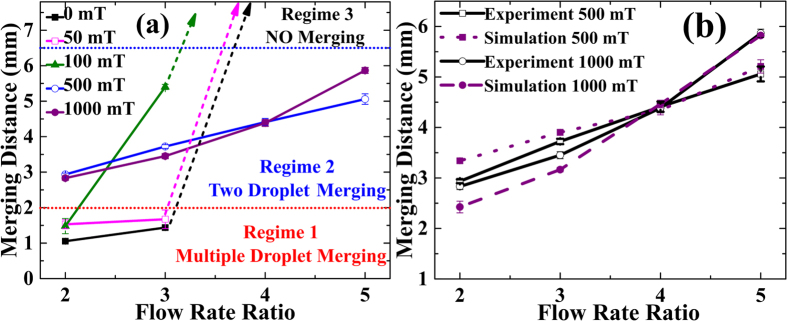
Variation in droplet merging distance Lm with increasing flow rate ratio Qr under a uniform magnetic field H (**a**) Experimental results for H = 0, 50, 100, 500, 1000 mT. (**b**) Experiments vs simulations at H = 500, 1000 mT. Please refer Table 2 for notation.

We have quantified droplet merging under the influence of a magnetic field through a study of the variation of merging length (Lm) with a magnetic field (H). The merging distance Lm is defined as the distance from the T-junction to the centre of the droplet at the point of droplet merging (Fig. 1a). Droplet merging under the influence of a magnetic field is the result of competition between the force due to fluidic pressure (which varies with flow rate ratio), surface tension force and magnetic volume force (**F**_**m**_)^75^. *Droplet merging will occur if the magnetic volume force exceeds the combined forces due to fluidic pressure and surface tension.* This competition divides droplet behaviour into three regimes (Fig. 3 and Fig. 4a). A video S2 is available in the supplementary files, showing the experimental droplet merging regimes:

- **Regime 1**: Multiple droplet merging, at magnetic field H = 0, 50 mT for Qr ≤ 3 and H = 100 mT for Qr = 2 (red filled squares in Fig. 3a, experimental micrograph of Fig. 3b and region below the red dotted line in Fig. 4a).
- **Regime 2**: Two droplet merging at H ≥ 500 mT, for all Qr (blue filled circles in Fig. 3a, experimental micrographs in Fig. 3c and region between red and blue dotted line in Fig. 4a).
- **Regime 3**: No merging, at Qr ≥ 4 for magnetic field H ≤ 100 mT (black filled triangles in Fig. 3a, experimental micrograph of Fig. 3d and region above blue dotted line in Fig. 4a, denoted by dashed arrow lines).

#### Regime 1 and Regime 3

Regime 1, which is the region below the red dotted line in Fig. 4a, shows *multiple droplet merging* of droplets (Fig. 3b). This multiple droplet merging leads to the unrestricted merging of more than two droplets. It is caused by close spacing of ferrofluid droplets at low flow rate ratios, viz. Qr = 2, 3 for H less than 100 mT. Two droplet merging was observed at 100 mT for flow rate ratio of 3 (Fig. 3a). The other extreme is regime 3, i.e., the *no merging regime* (Fig. 3d), which is shown by dashed arrow lines in Fig. 4a. This corresponds to a larger merging distance (Lm ≥ 6.5 mm) for Qr greater than 3 and H less than 500 mT (Fig. 3a). The fluidic pressure force at higher flow rate ratios (Qr = 3, 4) dominates regime 3.

#### Regime 2 (Two Droplet Merging)

Two droplets merging (Fig. 3c) is shown in regime 2 of Fig. 4a, which is the region between the two horizontal (red and blue) dotted lines. The merging distance Lm increases linearly with increasing flow rate ratio at H = 500, 1000 mT. The magnetic volume force due to magnetic field ≥500mT dominates at all Qr. Two droplet merging was observed at 500 mT and 1000 mT at all Qr (Fig. 3a). Two droplet merging was also observed at 100 mT for Qr = 3 (Fig. 3a). The merging distance is larger at Q4H1000 than that for Q4H500, which may be due to increased droplet velocity for the set Q4H1000 (Fig. 4a). A linear behaviour of droplet merging distance with flow rate ratio was observed (Fig. 4a).

#### Transitions between various regimes

The transitions between various regimes can be explained by the competition between three forces: (i) hydrodynamic force (ii) magnetic volume force and (iii) surface tension force. The merging length, Lm was used to quantify these transitions: (i) if Lm ≤ 2 mm results in multiple droplet merging, which indicates low hydrodynamic force (resulting in close spacing) and insufficient magnetic volume force. (ii) Lm > 6.5 mm, indicating high hydrodynamic force (resulting in greater droplet spacing), dominating over magnetic volume force. (iii) 2 mm <Lm ≤6mm leads to controlled merging due to the dominating effect of magnetic volume force over both hydrodynamic and surface tension force.

### Simulation of Dependence of Lm on Interfacial tension

At zero magnetic field, the ferrofluid droplets are spherical to minimize surface area i.e., minimum ferrofluid-oil interfacial tension. Under the influence of a *uniform magnetic field,* the ferrofluid droplet *elongates*, deviating from its spherical shape. With increasing magnetic field, the elongation of ferrofluid droplet increases, implying increased ferrofluid-oil interfacial tension^73^,^74^,^76^.

**Figure 5:**
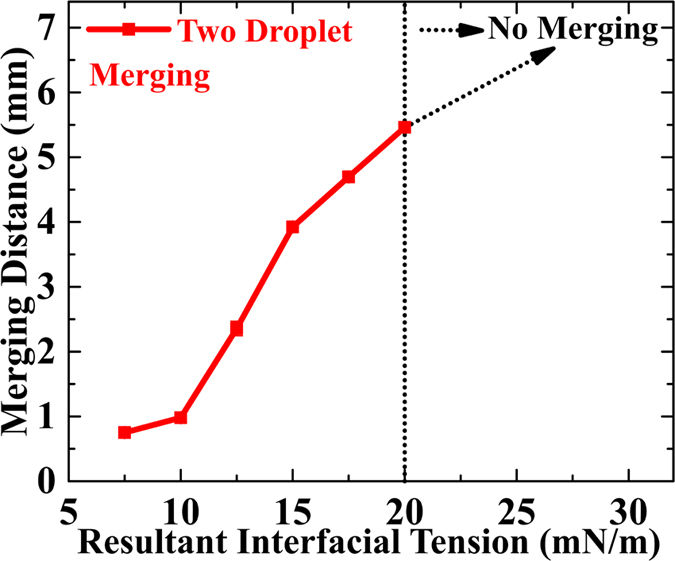
Simulations of the dependence of merging distance on the *resultant interfacial tension* at magnetic field H = 500 mT and flow rate ratio Qr = 3. At RIT ≤ 20 mN/m merging was observed. A supplementary info file and video S2 are available in supplementary information, showing the simulated droplet merging.

Moving ferrofluid droplets in a magnetic field exhibit *pairing of ferrofluid droplets* before merging. The ferrofluid droplets attract each other, form pairs, travel some distance together, and then merge to form a single droplet (please see video S2 in supplementary information). This process depends on (i) uniform magnetic field strength, (ii) volume of ferrofluid droplet, (iii) flow rates of CP, DP, and (iv) interfacial interactions. Interfacial interactions are due to (i) the surfactants present with the system and (iii) increased ferrofluid-oil interfacial tension due to droplet elongation in the magnetic field. We used a *resultant interfacial tension* (RIT) between the ferrofluid and the silicone oil to calculate the droplet merging distance in our simulations (please see supplementary info file and video S2 for the simulated droplet merging, available in the supplementary information). The droplet merging distance is a function of magnetic field strength, *resultant interfacial tension* and flow rates of CP, DP. Figure 5 shows the variation in droplet merging distance with RIT at a magnetic field of 500 mT and flow rate ratio of 3. The merging distance increases slowly for RIT ≤10 mN/m and rapidly for RIT greater than 10 mN/m. For RIT greater than 20 mN/m no merging was observed, indicating that interfacial forces dominate over the magnetic field. The modelled merging distance at RIT of 15 mN/m matches with an experimental value for Qr = 3 and H = 500mT (Fig. 4b). This value is higher than the value of 12 mN/m for ferrofluid-silicone oil interfacial tension without magnetic field^64^.

### Experimental and Simulation Results of Droplet Merging at H ≥500 mT

Figure 4b shows the experimental and simulation results for the variation of merging distance with increasing flow rate ratio. These micromagnetofluidic numerical simulations were performed for regime 2, which corresponds to the magnetic field induced merging at 500 mT and 1000 mT. Evidently, from Fig. 4b, the simulation, and experimental results are in good agreement. However, slight deviation was observed at lower flow rate ratios for H = 500, 1000 mT, which shows a linear variation compared to our experimental results. The observed mismatch may be due to non-linear effects in our experimental flow rates, which become significant at lower flow rate ratios compared to the magnetic volume force.

### Process of Droplet Merging

**Figure 6:**
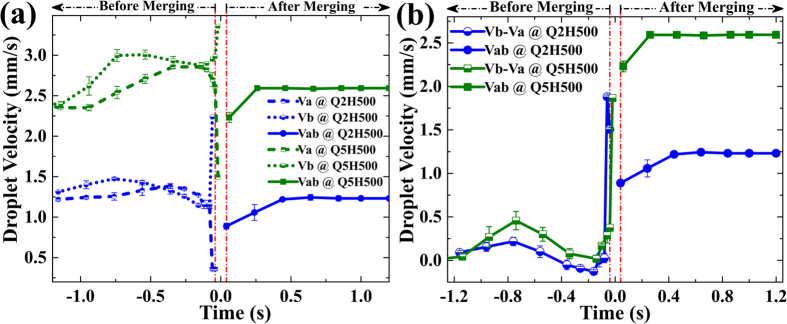
Experimental variation in droplet velocity at H = 500 mT for Q2 and Q5. (**a**) Velocity variation before merging (Va, Vb) and after merging (Vab). (**b**) Variation in differential velocity Vb-Va with time, implying necessary condition for droplet merging of Vb-Va > 0 (Please refer Table 2 for notation).

**Figure 7:**
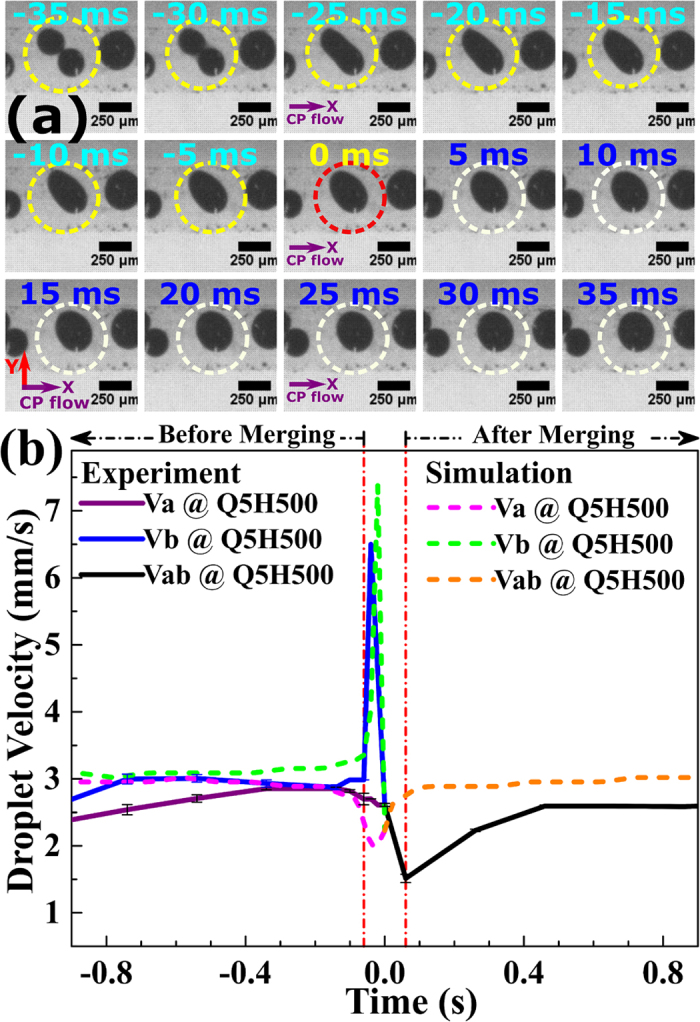
Time variation of droplet velocity during merging at H = 500 mT and flow rate ratio Q5. (**a**) Experimental micrograph of DMR, states of the droplet merging with respect to time are encircled, with TMP at 0 ms. (**b**) Simulations vs Experiments (Please refer Table 2 for notation). The purple arrow indicates the direction of the CP flow (x-direction). The magnetic field is in the y-direction.

To study the process of droplet merging, we investigated the variation in droplet velocity with time at a constant flow rate ratio and H = 500 mT, for sets Q2H500 and Q5H500 (Table 2). Two droplets travelling with velocity Va and Vb were assumed (Fig. 1a): Va indicates the velocity of droplet a, which enters first in the microchannel, and Vb is the velocity of the droplet b, which follows droplet a. Vab is the velocity of the merged droplet ab (Fig. 1a). Figure 6 shows the variation in droplet velocity with time. Negative times indicates velocity variation before merging, positive time indicates velocity variation after merging. The region between the two vertical dash-dotted red lines indicates the droplets merging region (DMR) (Fig. 6 and Fig. 7). Before DMR, droplets exist in the non-merged state; after DMR the droplets have merged and travel as a single entity. The transition merging point (TMP) was assigned time equal to 0s. The merged interface length of the droplets a and b is denoted by MIL. A value of MIL greater than half of the droplet circumference (HDC) denotes complete merging of the droplets. High-speed micrographs of the DMR revealed various states of droplet merging with time, t (Fig. 7a) viz. (i) at time of −40 ms, droplet interfaces come in contact (ii) MIL is less than HDC [for time <0ms] (iii) MIL is equal to HDC [for time = 0 s] (iv) MIL is greater than HDC [for time >0ms] (v) complete merging of the droplet interfaces, at the end of DMR [for time >0 ms] corresponds to a minimum velocity Vab (Fig. 6 and Fig. 7b).

Assuming that the direction of CP flow is the positive direction of velocity, there are three possible cases with respect to the variation of droplet velocity Va and Vb with time, which result in merging of two moving droplets: (i) Va, Vb travelling in the flow direction [Vb > Va] (ii) Va and Vb travelling in the opposite flow direction [-Va > -Vb] (iii) both droplets travel towards each other. The required condition for droplet merging, that the differential velocity, Vb-Va should be positive. It is evident from Fig. 6b that near DMR, the differential velocity increases, satisfying Vb-Va > 0 for both sets Q2H500 and Q5H500. At TMP, Vb-Va reaches to a maximum value, resulting in the merging of droplet a and droplet b.

It is interesting to note that our simulation results for the time variation of velocity are in close agreement with experiments (Fig. 7b). The droplet velocity Va decreases and Vb increases for both experiments (Fig. 6a and Fig. 7b) and simulations (Fig. 7b). Close to the TMP, the droplet velocity Vb attains a maximum value, which was also observed in our simulations. The simulated Vb was found to be slightly higher than the experimental Vb. At TMP, the droplets merge and beyond TMP, the merged droplet travels with a velocity Vab, which initially falls (Fig. 7b). Vab increases beyond DMR and then saturates. The simulated droplet velocity profile Vab was found to be slightly higher than the experimental Vab (Fig. 7b). This mismatch may be due to the continuum approximation used in our modelling, which does not consider interparticle interactions of magnetic particles.

### Application of Magnetically Induced Merging

**Figure 8:**
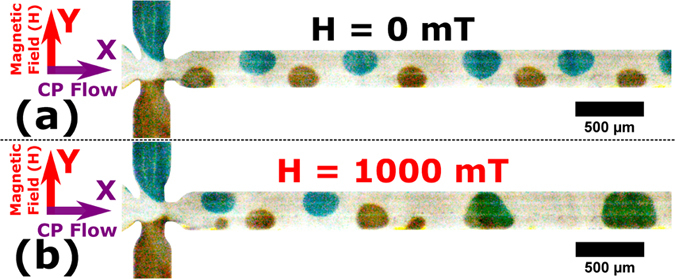
Magnetically induced merging of composite droplets (**a**) No merging at H = 0 mT (**b**) Merged green droplets at H = 1000 mT. The CP flow is along the x-direction. The magnetic field is in the y-direction. Composite droplets were generated at flow rates of (i) CP = 120 μl/h (ii) DP1 (blue droplets) = 20 μl/h (iii) DP2 (yellow droplets) = 10 μl/h.

Merging and mixing of droplets on a LoC platform was demonstrated using colour dye + magnetite (ferrofluid) composite droplets (Fig. 1b and Fig. 8). Two composite droplets were used, both containing 10% loading of ferrofluid in (i) 10% (v/v) blue dye in deionized water (ii) yellow dye solution. A schematic of the experiment is shown in Fig. 1b. These composite droplets were generated at flow rates of (i) CP = 120 μl/h (ii) DP1 (blue droplets) = 20 μl/h (iii) DP2 (yellow droplets) = 10 μl/h. Evidently (Fig. 8), no merging occurs when the magnetic field was not applied. When a magnetic field of 1000 mT was applied blue droplets merge and mix with yellow droplets, yielding green droplets. The mixing of blue and yellow composite magnetic droplet is evident from the green colour (Fig. 8).

## Conclusions

Droplet merging in a Lab-on-a-Chip environment under the influence of a *uniform* magnetic field was investigated. The effect of applied magnetic field and flow rate ratio was investigated. Control of the droplet merging distance Lm was demonstrated, Lm increases with increasing flow rate ratio. A droplet micro-magnetofluidic model was developed, our numerical results are in good agreement with experimental results. The condition for droplet merging was found to be that the differential velocity of two droplets a and b, Vb-Va should be positive; this was verified experimentally and numerically. The process of droplet merging was studied through variation of the droplet velocity with time under the influence of a uniform magnetic field. The merging and mixing of composite magnetic droplets was demonstrated under the influence of a magnetic field. The present work is useful for wireless, programmable merging and mixing of droplets on a LoC platform, which finds applications in biosensing, bioassay, microfluidic-based synthesis, reaction kinetics, and magnetochemistry.

### Methodology

Details of materials used in the experiments, the microfluidic chip, our experimental setup, parameters, physics of the numerical approach and simulation methodology are summarized in this section.

#### Materials

We used two immiscible phases for the generation of droplets: silicone oil (KF96A-100CS, Shin-Etsu, Japan) as CP and water-based ferrofluid EMG 807 (Ferrotec, Singapore) as DP. We added a small amount of surfactant 0.3% (v/v) Tween 20 (Sigma-Aldrich, Singapore) to the DP for uniformly sized droplet generation. The measured viscosities (Brookfield rheometer, model: LV-DV3T) of silicone oil and ferrofluid (with surfactant) were 105 ± 0.10 mPa.s and 1.50 ± 0.01 mPa.s, respectively. The measured densities^77^ of silicone oil and ferrofluid were 0.965 ± 0.005 × 10^3^ kg/m^3^ and 1.100 ± 0.004 × 10^3^ kg/m^3^, respectively. The properties of the water-based ferrofluid EMG 807 are summarized in Table 1.

We used yellow and blue food dyes (Star Brand food colour, FairPrice, Singapore) to visually demonstrate merging and mixing. 10% (v/v) blue food dye solution was prepared in deionized (DI) water. Yellow food dye solution was used without dilution. 10% EMG 807 ferrofluid was mixed in both solutions, along with 0.5% (v/v) Tween 20 for uniform droplet generation.

#### Microfluidic Chip

Poly(methyl methacrylate) (PMMA) microfluidic chips, fabricated by a micro milling technique were used. Microfluidic chips were bonded using a thermal bonding technique (Specac, model: Atlas 15T, with heating assembly). We performed low-temperature thermal bonding^78^ (below the glass transition temperature of 105 °C for PMMA) to reduce channel deformation, at optimized parameters of the temperature of 95 °C, duration of 16 min and load of 50 kg. A dedicated chip holder was used to connect the tubing with the bonded microfluidic chip. AutoCAD 2015 was used to design the microfluidic chip with 75 mm in length and 25 mm in width. We used two different chip designs for the experiments (Fig. 1).

For ferrofluid droplet merging, a microfluidic chip with two inlets and one outlet was used (Fig. 1a). One inlet microchannel was used for CP and other for DP, each with 500μm in width and 150 μm in height. A T-junction with a cross-section of 150 × 150 μm^2^ (width × height) was used for droplet generation. The outlet microchannel was 500 μm in width and 150 μm in height. For colour dye droplet merging, a cross-junction chip was used to simultaneously generate two droplets with blue and yellow colour (Fig. 1b). The middle inlet was used for the CP (silicone oil), DP1 was used for blue, and DP2 for yellow colour dye droplets. Flow rates and flow rate ratios were tuned to obtain uniformly sized droplet generation. Chip dimensions are described in Fig. 1b.

#### Experimental setup

The micromagnetofluidic setup^68^,^69^ used for our experimental studies (Fig. 1c), consists of (i) microfluidic droplet generation, (ii) uniform magnetic field and (iii) high-speed imaging. A microfluidic droplet generation unit, consisting of a KDS Gemini 88 dual rate syringe pump connected to a microfluidic chip was employed. 2.5 ml Exmire Luer lock gastight syringes were used for fluid injection. IDEX tubing (0.50 mm inner diameter, 1.59 mm outer diameter) was used to connect the syringe to the microfluidic chip.

The uniform magnetic field H was generated by a water cooled DEXING electromagnet system (model: DXSB-178) (uniformity of ±0.1% for a region of 5 mm wide × 10 mm long), an air gap of 4 cm between the pole pieces was used. The magnetic field strength was controlled by tuning the current supplied to the electromagnet and maintained by a feedback loop through a Hall probe. The microfluidic chip was mounted in the middle of the pole pieces such that the magnetic field is perpendicular to droplet flow.

A Phantom Miro Camera (Model: M320s) coupled with a high magnification optics (Navitar Zoom 6000) was used for high-speed imaging. Videos are recorded at ~100 fps and ~200 fps (resolution 640 × 1200 pixels). ImageJ and Phantom Camera Control (PCC) software were used for image processing and analysis. Ferrofluid droplet size (Fig. 2) was measured by ImageJ software. The PCC software was used to determine the velocity of 10 consecutive ferrofluid droplets. Points in all graphs denote the mean of the measurements and the error bar represents the standard error of the mean^79^ for the corresponding data points.

#### Experimental Parameters

Droplet merging was investigated at four flow rate ratios, Qr = 2, 3, 4, 5 and at different uniform magnetic field strengths, H = 0, 50, 100, 500, 1000 mT. Sets and notations are summarized in Table 2.

#### Physics of numerical approach

Ferrofluids are suspensions of ferromagnetic nanoparticles in a carrier fluid stabilized by a surfactant to minimize agglomeration and sedimentation. They are sometimes referred as “Liquid magnets”. Ferrofluid droplets exhibit a strong, attractive force towards an applied magnetic field which depends on the strength and type of magnetic field (uniform or gradient)^80^. Rosensweig^80^ used a continuum model to explain the phenomena observed for ferrofluids, which can be extended to the case of moving ferrofluid droplets. Our micromagnetofluidic, 2D numerical model was developed for the case of ferrofluid droplets moving along the positive X-axis direction and uniform magnetic field applied in the positive Y-direction (Fig. 1a). The 2D approximation is valid for the geometry used for the experiments.

#### Equation of motion

The equation of motion for ferrofluid droplets under the influence of force **F**_**m**_ is given by^71^,^75^,^80^,


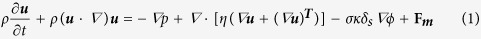



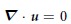


Where, **F**_**m**_ is the magnetic volume force, **u** is velocity, p is pressure, σ is surface tension, κ is curvature and δ_s_ is the smoothed delta function which is zero everywhere except at the interface and ϕ is the level set function^68^,^75^,^80^. The density ρ and viscosity η are defined using the weighted average interpolation, for a volume fraction 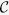 of the ferrofluid (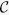 = 1 represents DP) by the following equations^68^,^69^.


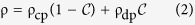



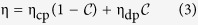


#### Ferrofluid magnetization

At large magnetic fields, magnetization of the ferrofluid follows a non-linear behaviour, which is taken into account by the Langevin function. The ferrofluid magnetization at various magnetic fields was defined using the Langevin function L(γH)^65^,^80^ as,


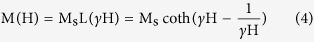


Where, γ = (3χ_o_/M_s_), χ_o_ is initial magnetic susceptibility and M_s_ is saturation magnetization of the ferrofluid.

The magnetic susceptibility at magnetic field H then takes the form^65^,^80^,


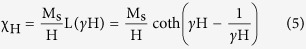


#### Magnetic volume force

The magnetic field was defined using Maxwell’s equation for magnetostatics^80^, as below.


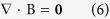



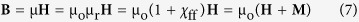


Where μ = μ_o_μ_r_ is the permeability and μ_r_ = (1 + χ_ff_) is the relative permeability of the ferrofluid.

The merging behaviour of the ferrofluid droplets was defined by considering the magnetic volume force: (i) **F**_**m1**_: acting at the interface of ferrofluid droplet and silicone oil. This force is responsible for the deformation of the ferrofluid droplets in a uniform magnetic field. (ii) **F**_**m2**_: acting on the total volume of the ferrofluid droplets.

1. Magnetic Volume Force F_m1_. We used the magnetic stress tensor formulation to calculate the magnetic force **F**_**m1**_^71^,^80^. The magnetic stress tensor for an applied magnetic field **H** in indicial form is given^80^ by,


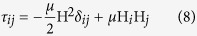


The magnetic body force **F**_**m1**_ is then defined by the following equation^80^


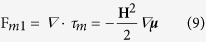


This force only acts on the interface, which is taken into account by the smoothed delta function (δ_s_)^71^,^76^.


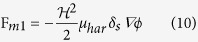


The magnetic permeability of the medium was determined by the harmonic mean (μ_har_)^64^,^74^:


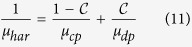


where, μ_cp_ ~ μ_0_ and μ_dp_ = (1 + χ_H_). Evidently from the above equation, the value of magnetic permeability μ_har_ is equal to the magnetic permeability of the ferrofluid inside the droplet (for 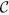 = 1) and outside it is equal to the magnetic permeability of oil (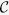 = 0).

2. Magnetic Volume Force F_m2_. The magnetic volume force, F_m2_ acts on the total volume of the ferrofluid droplet, it defines the motion of ferrofluid droplets in the microchannel under the influence of a magnetic field H^69^,^82^. This force depends on the total volume of the ferrofluid droplet. The magnetic volume force F_m2_ was defined by the following equation, for ferrofluid volume fraction 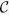 and susceptibility χ_H_.


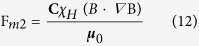


#### Simulation

We developed a droplet micro-magnetofluidic numerical model using the above constitutive relations^71^. Numerical simulations were performed by COMSOL Multiphysics software, using the laminar two-phase flow, a level set method in fluid dynamics module. The uniform magnetic field was modelled with no currents method in AC/DC module^83^. Extra-fine meshing was performed for whole geometry with 34046 total elements. The meshing of the microchannel domain was created using 20066 triangular elements with a size of 0.25 μm (minimum) to 21.7 μm (maximum). Meshing for magnetic field domain was created by 14265 triangular elements of size 1.1 μm (minimum) to 150 μm (maximum).

Interestingly, our micro-magnetofluidic numerical model simulates the: (i) generation of ferrofluid droplets according to the experimental flow rates and flow rate ratios under the influence of magnetic field (ii) deformation of the droplets in a uniform magnetic field (iii) magnetically induced merging of ferrofluid droplets (please see video S2 in supplementary information). Droplet generation, deformation, and consequent merging show a dependence on the interfacial tension between the ferrofluid and silicone oil. At zero magnetic field, the interfacial tension between silicone oil and ferrofluid was 12 mN/m^64^, the effective interfacial tension changes with magnetic field^73^,^74^,^76^. The magnetic volume force **F**_**m1**_ acting on the interface changes the interfacial tension, leading to droplet deformation, which was evident from our experimental results and our numerical simulations. From our simulated results, the dependence of droplet merging on interfacial tension was determined (Fig. 5).

## Additional Information

**How to cite this article**: Varma, V. B. *et al*. Droplet Merging on a Lab-on-a-Chip Platform by Uniform Magnetic Fields. *Sci. Rep.* **6**, 37671; doi: 10.1038/srep37671 (2016).

**Publisher's note:** Springer Nature remains neutral with regard to jurisdictional claims in published maps and institutional affiliations.

## Supplementary Material

Supplementary Information

Supplementary Video S2

## Figures and Tables

**Table 1:** Properties of EMG 807 ferrofluid (dispersed phase).

Quantity	Notation	Values
^#^Viscosity (mPa·s)	η_dp_	1.50 ± 0.01
^#^Density at 25 °C (×10^3^kg/m^3^)	ρ_dp_	1.100 ± 0.004
^*^Saturation Magnetization (mT)	M_s_	11
^*^Initial Magnetic Susceptibility (SI units)	χ_o_	1.88
^*^Magnetic Particle Concentration (% vol)	c_v_	2
^*^Particle Diameter (nm)	d_p_	10

Note: ^*^Properties as provided from supplier data sheet (Ferrotec Singapore), ^#^Measured properties.

**Table 2:** Sets and notations (Silicone oil was used as continuous phase (CP) and ferrofluid was used as dispersed phase (DP)).

Set	Flow rate ratio			Sets at an applied uniform magnetic field, H (mT)				
	Qr	CP (μl/h)	DP (μl/h)	0	50	100	500	1000
Q2	2	200	100	Q2H0	Q2H50	Q2H100	Q2H500	Q2H1000
Q3	3	300	100	Q3H0	Q3H50	Q3H100	Q3H500	Q3H1000
Q4	4	400	100	Q4H0	Q4H50	Q4H100	Q4H500	Q4H1000
Q5	5	500	100	Q5H0	Q5H50	Q5H100	Q5H500	Q5H1000

Where, Qr: flow rate ratio = Q_cp_/Q_dp_. (Refer Table 1 for CP, DP properties).
