# Supplementary material for: Droplet Merging on a Lab-on-a-Chip Platform by Uniform Magnetic Fields
Source: Sci Rep. 2016 Nov 28;6:37671. doi: 10.1038/srep37671 (PMC5124862; doi:10.1038/srep37671)
Supplement: Supplementary Information [file srep37671-s1.pdf]

Supplementary Information

Droplet Merging on a Lab-on-a-Chip Platform by Uniform Magnetic Fields

V. B. Varma[1+], A. Ray[1], Z. M. Wang[1], Z. P. Wang[2] and R. V. Ramanujan[1*+]

[1]*School of Materials Science and Engineering, Nanyang Technological University, Singapore, 639798.*

[2]*Singapore Institute of Manufacturing Technology, 71 Nanyang Dr, Singapore 638075.*

[*]Corresponding Author Electronic mail: ramanujan@ntu.edu.sg

---

Content:

S1. Simulation Results of Dependence of Lm on Resultant Interfacial Tension

S2. Video of Experimental and Simulated Droplet Merging on Lab-on-a-Chip Platform by Uniform Magnetic Fields

---

S 1. Simulation Results of Dependence of Lm on Resultant Interfacial Tension

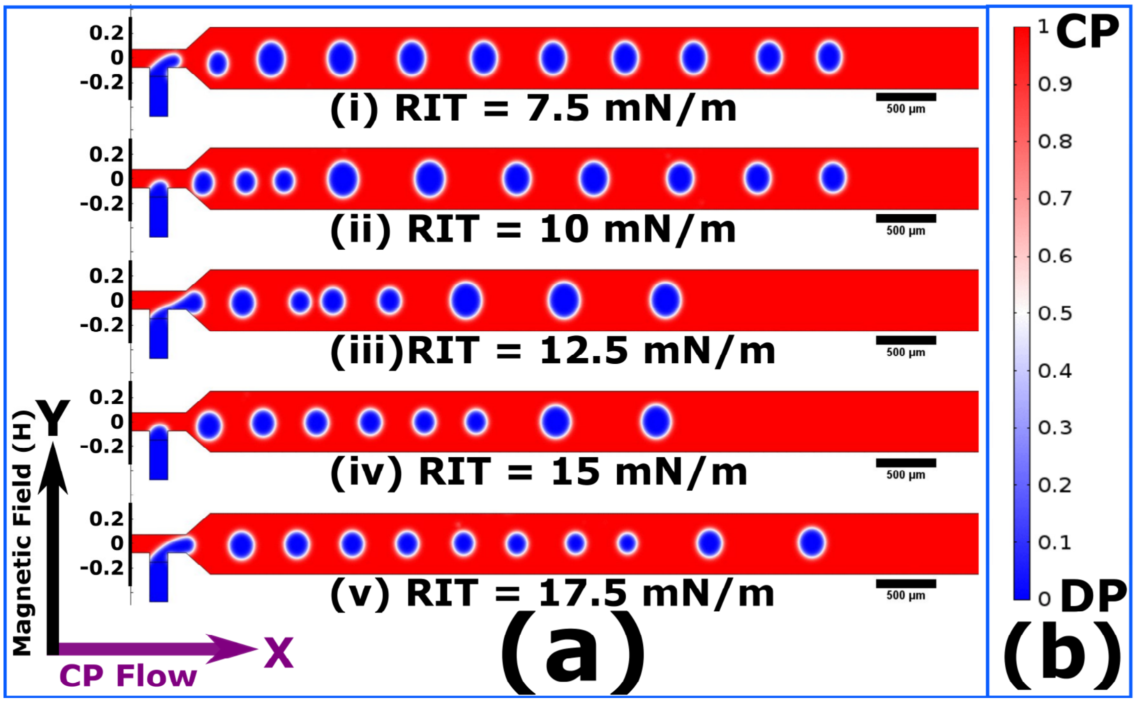

Supplementary Figure 1: Simulation results **(a)** The dependence of droplet merging distance Lm on *resultant interfacial tension* (RIT) at magnetic field H=500 mT (in the y-direction) and flow rate ratio Qr=3. The flow of the CP was in the x-direction. The scale is in mm. Scale bar=500 µm. (b) Colour bar for supplementary Figure1(a). Where, CP (red colour) and DP (blue colour) denote the continuous phase of oil and the dispersed phase of the ferrofluid, respectively. At RIT≤20 mN/m merging was observed. Droplet merging distance, Lm increases with increasing RIT value.

S 2. Video of Experimental and Simulated Droplet Merging on a Lab-on-a-Chip Platform by Uniform Magnetic Fields

Video Legend:

S2

Video Title:

Droplet Merging on a Lab-on-a-Chip Platform by Uniform Magnetic Fields

Video Description:

As shown in the video:

(i) When the magnetic field was not applied, no droplet merging occurs at flow rate ratio of 2 (Q2) and multi-droplet merging occurs at flow rate ratio of 5 (Q5).

(ii) When a uniform magnetic field H=500 mT was applied, Two Droplet Merging (TDM) occurs at both flow rate ratios, Q2 and Q5. Droplet merging length (Lm) is controlled by the flow rate ratios and applied magnetic fields.

(iii) The droplet merging length, Lm also shows a dependence on the ferrofluid-oil *resultant interfacial tension* (RIT). The simulation results demonstrate the increasing merging length Lm, with the increasing RIT values, at a constant flow rate ratio of 3 (Q3) and an applied magnetic field, H=500 mT.
